# The role of salinity in recovery of white sturgeon (*Acipenser transmontanus*) from stimulated angling stress

**DOI:** 10.1093/conphys/coad009

**Published:** 2023-03-17

**Authors:** Ryan B Shartau, Jacelyn Shu, Daniel W Baker

**Affiliations:** Department of Biology, The University of Texas at Tyler, 3900 University Blvd., Tyler, TX, USA, 75799; Department of Zoology, University of British Columbia, 4200 - 6270 University Blvd., Vancouver, British Columbia, Canada V6T 1Z4; Department of Fisheries and Aquaculture, Vancouver Island University, 900 Fifth Street, Nanaimo, British Columbia, Canada, V9R 5S5

**Keywords:** sturgeon, salinity, fish, exercise, angling stress, Acid–base regulation

## Abstract

White sturgeon (*Acipenser transmontanus*) in the Lower Fraser River are the focus of a catch-and-release angling fishery in British Columbia, Canada. However, the lower region of the catch area includes areas where tidal waters invade, and the consequence of salinity levels on recovery from an angling challenge are not characterized in sturgeon, despite theoretical implications of its import. We acclimated white sturgeon to various salinities (0, 10 and 20‰ (parts per thousand)) to investigate the effects of acclimation on recovery from stimulated angling stress that was induced through manual chasing. This challenge elicited the traditional physiological responses such as ion homeostasis disturbance, increases in secondary stress indicators and metabolic acidosis; however, environmental salinity altered the timing of recovery in some of the parameters measured. In addition, the severity of the intracellular pH disturbance in both heart and red blood cell seemed to be mediated in fresh water, yet the recovery pattern of plasma chloride and bicarbonate ions seemed to be facilitated by higher salinity. In general, responses were similar but not identical, leading us to conclude that the role of salinity on recovery from exercise is complex but not insignificant. Salinity may be important to behaviours exhibited by white sturgeon (such as migrations) in their respective saline environments, but less so around the impact of an angling stressor. Further exploration of this response may provide insight on whether the current tidal boundaries for angling white sturgeon are appropriate.

## Teaser

White sturgeon (*Acipenser transmontanus*) may be acclimated to different salinities in the Fraser River, which may impact their ability to recover from severe exercise induced by angling. Sturgeon acclimated to different salinities and subjected to stimulated angling stress showed differences in recovery rate of physiological parameters.

## Introduction

White sturgeon (WS) *Acipenser transmontanus* (Richardson, 1863) is the largest freshwater fish species in North America. They are believed to have existed as a species for >46 million y demonstrating their resilience to major geological and climatic changes over this time (Shedko, 2022). Unfortunately, due to human activities associated with overharvesting, pollution, dam construction and habitat destruction over the past 150 y, they are now endangered or threatened throughout much of their historical range (Hildebrand *et al.,* 2016). In British Columbia, the lower and middle Fraser River contains a population of WS of approximately 44 000 fish (Nelson *et al.,* 2019), which supports a year-round catch and release (C&R) fishery that draws considerable tourism revenue (Glova *et al.,* 2009). Although WS are the dominant sturgeon species in the Fraser River, green sturgeon (*A. medirostris*) are known to enter the lower Fraser River infrequently from the Salish Sea but are not a target for the C&R fisheries, and reports of capture in fresh water (FW) are rare (COSEWIC, 2004). Despite WS being heavily managed, there is long-term decline with changes to population structure that include fewer younger fish due to limited recruitment (Nelson *et al.,* 2013, 2019).

Primarily due to conservation concerns of this iconic fish, there have been numerous studies investigating the impact of C&R on WS populations (McLean *et al.,* 2016, 2019, 2020). Experimental work suggests that WS recover quickly from C&R angling in general and seldom experience mortality (McLean *et al.,* 2016, 2019, 2020), with an estimated direct mortality of angled fish <0.012% and 3-d postangling mortality rate of 2.6% (Robichaud *et al.,* 2006). Angling events can vary, with fight times ranging from seconds to >2 h with a median time of 5.78 min (McLean *et al.,* 2016), and WS are likely captured multiple times over their life. During capture events, WS often spend time out of water; however, they are tolerant of air exposure provided their gills are kept moist, as Shartau *et al*. (2017) found WS could recover from a 45-min air exposure.

The physiological challenges associated with C&R are largely due to stress related to exhaustive exercise during angling capture as fish struggle (Wood *et al.,* 1983). Exhaustive exercise in WS occurs while fish attempt to avoid capture, inducing a severe metabolic acid–base disturbance whereby large reductions in blood and red blood cell (RBC) pH are observed, along with smaller but significant reductions in tissue pH (Shartau *et al.,* 2017). This response is a well-documented pattern in fishes (Randall and Brauner, 1991; Nelson *et al.,* 1996; Harter *et al.,* 2014). The tolerance of WS to the severe acid–base disturbance associated with exercise likely accounts for the limited mortality observed during C&R because severe acidification of white muscle is implicated as a likely cause of death in fish (Wood *et al.,* 1983). The effect of exercise on sturgeon is altered by various factors including water temperature, exercise duration and intensity, as well as fish age, sex and life-stage (McLean *et al.,* 2016, 2020), making experimental exploration challenging.

Fishing for WS on the lower Fraser River occurs in both the nontidal freshwater (FW) region and the higher salinity tidal region, which extends >20 km upstream; in fact, the tidal effect can reach as far upstream as 84 km (Murray, 2016; Leung *et al.,* 2018). This salt wedge creates a stable region of higher salinity due to the higher density of salt water and may result in WS being acclimated to salinities ranging from FW up to 20‰ (parts per thousand), which occurs at the mouth of the river. Although WS can tolerate salinity (McEnroe and Cech, 1985; Vaz *et al.,* 2015), there may be physiological differences in the response during, and after, C&R fishing due to the physiological differences of fish acclimated to different salinities. Differences in salinity can elicit changes in the physiological response of fishes (Byrne *et al.,* 1972; Redding and Schreck, 1983; Wood, 1991; Nelson *et al.,* 1996; Pedersen and Malte, 2004; Allen *et al.,* 2014; Christensen *et al.,* 2018), including those related to acid–base disturbances (Randall and Brauner, 1991), likely a result of the osmotic and ionic differences incurred by fish between FW and seawater (Evans *et al.,* 2005; Damsgaard *et al.,* 2020).

Bouts of exhaustive exercise, such as that arising from C&R i) increase blood and tissue lactate due to increased anaerobic metabolism, ii) induce a rapid reduction in blood pH (pH_e_) (referred to as a metabolic acidosis) and iii) changes plasma ions and hematocrit, which are associated with water imbalance and loss of ion homeostasis. In some species, a reduction in tissue pH accompanies the metabolic acidosis (Ferguson and Tufts, 1992; Pankhurst and Dedualj, 1994; Nelson *et al.,* 1996). Recovery from acid–base disturbances typically occurs over the following 8–48 h, although water composition may alter the severity and recovery profiles (Larsen and Jensen, 1997; Tovey and Brauner, 2017; Sackville *et al.,* 2018). Consequently, the capacity to recover from severe C&R-induced exercise may differ depending on water salinity. Both an increase in severity and a decrease in recovery rate of the associated acidosis could induce exercised-induced mortality. As in trout, mortality has been linked to the large acid load incurred during exhaustive exercise and their inability to successfully compensate for it through typical pH regulatory mechanisms (Wood *et al.,* 1983).

Because WS from the lower Fraser River may be acclimated to different salinities, they may also experience different physiological responses as a result of C&R; these differences may translate into more severe outcomes for both short-term and long-term survival. In addition, because exhaustive exercise is a highly stressful event (Baker *et al.,* 2005b; McLean *et al.,* 2016), there are elevations in stress indicators, such as plasma cortisol and glucose, which are known to have long-term effects on whole animal function (Faught *et al.,* 2016) and can have maternal effects in progeny (Faught and Vijayan, 2018). Consequently, C&R capture of gravid females under differing salinities, for example, could exacerbate reproductive effects and negatively impact recruitment in the Fraser River in unforeseen ways.

Although sturgeon demonstrate the capacity to tolerate exhaustive exercise in FW (Kieffer *et al.,* 2001; Baker *et al.,* 2005a; Shartau *et al.,* 2017; Brown and Kieffer, 2019; Penny and Kieffer, 2019; Kieffer and May, 2020), it remains unknown how quickly they are able to restore their acid–base status and it is uncertain if acclimation to higher salinities affects severity of, or recovery from, this challenge. To examine the effect of salinity on exhaustive exercise associated with C&R, we subjected WS to stimulated angling stress (SAS) after acclimation to either 0, 10 or 20‰ and assessed the effects using a suite of physiological indicators. These three salinity levels were chosen because they represent FW, estuarine and dilute seawater found in the Salish Sea near the mouth of the Fraser River (Murray, 2016; Leung *et al.,* 2018). These hyposaline, isosaline and hypersaline waters, respective to the fish, have significantly different driving forces for both ions and water movement. To further characterize the role of salinity on SAS, we assessed recovery from this challenge at both 4 and 8 h postexercise, within which time partial or complete recovery would generally be expected (e.g. Baker *et al.,* 2005b). This work was designed to illustrate whether environmental salinity (and presumably anadromy) may play an important role not only in the exercise-based behaviours of fish, such as predator avoidance, prey capture or migration, but also to provide insight into whether C&R for WS in saline waters elevates the risk of mortality.

## Methods

### Animal acquisition and holding

All experiments were performed at the International Center for Sturgeon Studies (ICSS) at Vancouver Island University (VIU). WS were reared from hatch at ICSS from wild captive brood stock from a single male:female cross, and maintained in large, indoor flow-through tanks (23 m^3^) in dechlorinated City of Nanaimo tap water [61 μmol l^−1^ Na^+^, 69 μmol l^−1^ Cl^−^ (City of Nanaimo, 2015), pH ~6.6–6.8 (Amiri *et al.,* 2009)] at ~15°C under a simulated natural photoperiod and were fed daily to satiation using a 24-h belt feeder based on aquaculture produced feeding tables (Conte *et al.,* 1988). WS (*n* = 78) used in this study were 3 y old and had a mean weight of 2.97 ± 0.06 kg. Food was withheld 48 h before manual chasing and experimental sampling. Experiment protocols were approved by the Vancouver Island University Animal Care Committee (Animal Usage Protocol: 2018-04-R).

### Salinity acclimation

WS were divided into three experimental groups (*n* = 26 per group) of different salinities (0, 10 and 20‰) in stand-alone tanks (1.8 × 1.8 × 0.7 m) with a total density per tank of approximately 33.5 kg m^−3^. The 0‰ treatment group was reared in dechlorinated City of Nanaimo tap water (as previously), whereas fish in the other two groups were created by adding calculated amounts of Instant Ocean® dissolved in that same water, increasing salinity 3–4‰ every 2 d until the desired salinity was reached. Aeration provided *via* an air compressor was provided within each tank to maintain dissolved oxygen at >80% of air saturation. Water changes of 25–50% were done as needed (roughly every 2 d) to keep ammonia levels below acceptable thresholds (~0.01 mg/L) (Meade, 1985). Sturgeon were fed 0.5% body weight daily (EWOS brood feed for salmonids—4 and 5 mm, 50% protein). Fish were acclimated to higher salinities for at least 2 wk before experimentation and showed no evidence of ill health associated with salinity acclimation. Experiments with all three salinities were run simultaneously (i.e. held at identical times in identical tanks at similar densities), with all sampling done through 4 d from 18–22 December 2018 to avoid seasonal effects.

### Stimulated angling stress and recovery

Sturgeon were removed from tanks and subjected to a two-step exhaustive exercise protocol previously used (Shartau *et al.,* 2017) to stimulate angling stress. Fish were chased with a plastic stick for 5 min or until the fish was completely exhausted, allowed to rest for 5 min, then chased again until complete exhaustion (i.e. no further escape response exhibited to being chased). Fish were then placed in individual aerated recovery tanks (1 × 0.5 × 0.5 m) matching the salinity of that of each group and allowed to recover for 0.5, 4 or 8 h. Control fish were sampled directly from the respective holding tanks.

### Blood sampling, tissue sampling and ions

After the appropriate recovery period, fish were rapidly removed from the recovery box and killed by cephalic concussion, after which blood (8 mL) was drawn caudally *via* a syringe (10-mL syringe, 23 G 3.1-cm needle) and placed on ice. After this procedure, complete hearts were removed within 2 min, gently squeezed and patted dry to remove blood, placed in aluminum foil and immediately placed in liquid N_2_ and then transferred to an ultracold freezer (−80°C) at the end of the day. Blood was divided into two aliquots. Blood pH, Hct, lactate and glucose were measured from one aliquot; the other aliquot was centrifuged for 3 min at 10 000 rpm and plasma was removed for measurement of total CO_2_ (TCO_2_), osmolarity and [Cl^−^].

Blood pH was measured using a glass electrode (Radiometer Analytical SAS pH electrode; GK2401C, Cedex, France or Orion 8302BNUMD ROSS Ultra pH/ATC Triode) connected to a pH meter (Orion Star A211 pH meter, ThermoFisher Scientific, Waltham, MA, USA). Intracellular pH (pH_i_) of heart tissue and RBC were measured using the same glass pH electrode previously described. RBC pH_i_ was measured using the freeze–thaw method as described and validated previously (Zeidler and Kim, 1977; Baker *et al.,* 2009). Tissue pH_i_ was measured using the metabolic inhibitor tissue homogenate method (MITH) and the pH electrode previously mentioned (Pörtner *et al.,* 1991; Baker *et al.,* 2009). Plasma TCO_2_ was measured using a TCO_2_ analyser (Corning model 965 Analyzer); the remaining plasma was used to measure [Cl^−^] ions (HBI model 4 425 000; digital chloridometer), osmolarity (model 5520; Westcor Vapor Pressure Osmometer), lactate (Lactate Pro™) and glucose (Onetouch™ Ultra2) (Baker *et al.,* 2005b).

### Calculations and statistical analysis

Plasma [HCO_3_^−^] and *P*CO_2_ were calculated using TCO_2_ and pH values as described by Brauner *et al*. (2004). CO_2_ solubility coefficient and the logarithmic acid dissociation constant (pK’) for plasma were determined based on previous work by Boutilier *et al.* (1984). All values are expressed as mean ± SEM throughout; *n* = 8 for all treatments except where otherwise noted. Data were compared by Welch *t* test or, where multiple treatments were evaluated, data were analysed by an analysis of variance (ANOVA), followed by Tukey *post hoc* test to compare all groups with each other. When two-way ANOVA interactive terms were significant, the factors were analysed separately using a one-way ANOVA. When data did not meet normality (Shapiro–Wilk normality test) or equal variance (Bartlett test) assumptions, a Kruskal–Wallis test followed by Dunn multiple comparison test was used (*p* < 0.05) to confirm conclusions. GraphPad Prism (v.8) was used for all statistical analyses and for preparation of figures.

## Results

**Figure 1:**
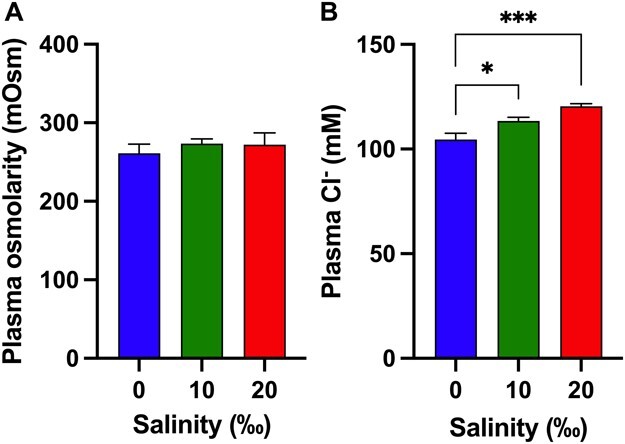
Plasma osmolarity (A) and chloride (B) in WS (*Acipenser transmontanus*) after 4 w in the respective salinities (‰). Error bars represent standard error about the mean. An asterisk indicates significant differences between groups as compared by the brackets ends.

As evident from the lack of differences amongst plasma osmolarity measurements from different salinities (Fig. 1A), WS exhibited complete acclimation to 10 and 20‰ over the time period allowed. Neither salinity exposure nor exhaustive exercise to simulate the stress of angling imposed by manual chasing resulted in mortality in any treatment group of animals.

### Osmoregulatory changes

Exposure to higher salinities did not significantly alter plasma osmolarity, although, as is typical in many marine fishes when compared with FW fishes (Edwards and Marshall, 2013), osmolarity of fish in 0‰ tended to be lower at 261 mOsM compared with 273 and 272 mOsM in 10 and 20‰, respectively (Fig. 1A). Overall, plasma osmolarity was significantly different among salinity (*F*_2,64_ = 9.49, *P* < 0.001), time (*F*_3,64_ = 19.68, *P* < 0.001), but not the interaction (Table 1), with a *post hoc* test revealing that plasma osmolarity was significantly higher in individuals at 0.5 h than control fish; osmolarity was no longer different than control fish after 8 h (Fig. 3C).

**Table 1:** Results from two-way ANOVA

**Parameter**	**Source of variation**	***SS***	***Df***	***MS***	***F***	***P***
**Blood pH**	Salinity	0.050	2	0.025	2.817	0.103
	Time	1.444	3	0.482	54.040	<0.001
	Interaction	0.100	6	0.016	1.851	0.067
	Residual	0.570	64	0.010		
**RBC pH**	Salinity	0.2123	2	0.166	10.190	0.0002
	Time	0.7369	3	0.2456	23.590	<0.0001
	Interaction	0.1813	6	0.0302	2.902	0.0149
	Residual	0.6352	61	0.0104		
**Heart pH**	Salinity	0.0515	2	0.0257	3.810	0.0275
	Time	0.0627	3	0.0209	3.095	0.0332
	Interaction	0.0389	6	0.0065	0.960	0.4596
	Residual	0.4186	62	0.00675		
**Plasma HCO**_**3**_^**−**^	Salinity	334.7	2	167.4	17.540	<0.0001
	Time	279.1	3	93.04	9.750	<0.0001
	Interaction	127.6	6	21.27	2.229	0.0519
	Residual	591.6	62	9.543		
**Plasma osmolarity**	Salinity	9106	2	4553	9.491	0.0002
	Time	28 324	3	9441	19.680	<0.0001
	Interaction	1431	6	238.5	0.497	0.8082
	Residual	30 701	64	479.7		
**Plasma lactate**	Salinity	27.59	2	13.79	2.382	0.1005
	Time	637.0	3	212.3	36.670	<0.0001
	Interaction	56.61	6	9.435	1.629	0.1535
	Residual	370.6	64	5.791		
**Hematocrit**	Salinity	45.13	2	22.56	3.379	0.0403
	Time	384.2	3	128.1	19.180	<0.0001
	Interaction	169.1	6	28.18	4.220	0.0012
	Residual	427.4	64	6.678		

Plasma [Cl^−^] differed between salinities (0‰—*F*_3,19_ = 3.429, *P* = 0.038; 10‰—H = 16.78 (2), *P* < 0.001; 20‰—*F*_3,22_ = 3.885, *P* < 0.05). Differences in plasma [Cl^−^] between salinities at each time point occurred at rest (*F*_2,14_ = 15.08, *P* < 0.001), 4 (*F*_2,16_ = 9.695, *P* < 0.001) and 8 h (H = 10.84 (3), *P* < 0.01); *post hoc* test revealed differences between 0‰, 10 and 20‰ at rest and at 4 h, whereas at 8 h post-SAS, differences were present between 10 and 20‰ (Figure 3D).

### Extracellular acid–base status

**Figure 2:**
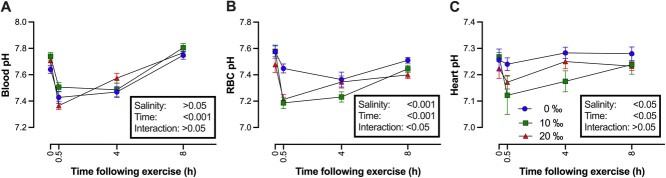
The effect of manual chasing on (A) blood pH, (B) red blood cell pH and (C) heart pH of 3-y-old WS (*Acipenser transmontanus*) acclimated to 0 (blue lines), 10 (green lines) or 20‰ (red lines) and sampled at 0.5, 4 or 8 h after the challenge. Error bars represent SEM.

**Figure 3:**
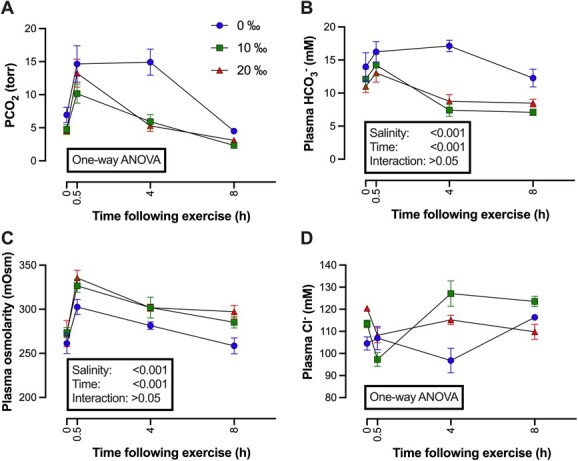
The effect of manual chasing on plasma (A) *P*CO_2_, (B) [HCO_3_^−^], (C) osmolarity and (D) [Cl^−^] in 3-year-old WS (*Acipenser transmontanus*) acclimated to 0 (blue lines), 10 (green lines) or 20‰ (red lines) and sampled at rest (0 h), 0.5, 4 or 8 h. Error bars represent SEM. See text for results of one-way ANOVA.

SAS resulted in changes to blood acid–base in all salinity groups (Fig. 2A). Blood pH (pH_e_) was reduced in all treatment groups 0.5 h postexercise. The mean pH_e_ across all salinities was reduced from 7.70 ± 0.02 to 7.44 ± 0.02 at 0.5 h, with pH_e_ values remaining depressed between 0.5 and 4 h postexercise, but almost identical to control values by 8 h in all three groups (Fig. 2A). Differences in pH_e_ occurred in the time after SAS (*F*_3,64_ = 54.04, *P* < 0.001) (Table 1); there was no effect of salinity on pH_e_ (*F*_2,64_ = 2.817, *P* = 0.067), with the exception of 0.5 h postexercise between the 10 and the 20‰ groups revealed by a *post hoc* test. The pattern of changes in blood *P*CO_2_ were similar to those of pH_e_, because values were elevated at 0.5 h postexercise in all salinity groups, returning to control values by 8 h (Fig. 3A). Kruskal–Wallis test revealed *P*CO_2_ changes after SAS were significant in all three salinities (0‰—H = 15.35 (2), *P* < 0.01; 10‰—H = 14.85 (2), *P* < 0.01; 20‰—H = 15.73 (2), *P* < 0.01), with *post hoc* test uncovering differences in *P*CO_2_ between 0.5 and 8 h at all three salinities. Differences in *P*CO_2_ between salinities at each time point occurred 4 (*F*_2,16_ = 16.37, *P* < 0.001) and 8 h (*F*_2,17_ = 12.19, *P* < 0.001) after SAS, with *post hoc* test indicating differences between 0, 10 and 20‰ (Fig. 3A). SAS-induced changes in plasma [HCO_3_^−^] with both interval postexercise (*F*_3,62_ = 9.75, *P* < 0.0001) and salinity (*F*_2,62_ = 17.54, *P* < 0.0001) having significant effects (Fig. 3B). A *post hoc* test indicated that 4 h postexercise, plasma [HCO_3_^−^] was significantly lower in the 10 and 20‰ groups both compared with the time-matched 0‰ group and the preceding 0.5 h postexercise group. Similarly, the *post hoc* test indicated that at 8 h, fish acclimated to 0‰ had [HCO_3_^−^] significantly elevated over that of fish acclimated to 10‰ (Fig. 3B).

### Intracellular pH

Salinity acclimation did not result in significant changes to RBC pH (*F*_2,12_ = 2.523, *P* = 0.12; Fig. 2B) (Table 1). After the angling challenge, RBC pH significantly changed in all salinities over the 8 h post-SAS (0‰—*F*_3,21_ = 4.12, *P* < 0.05; 10‰—*F*_3,20_ = 21.47, *P* < 0.001; 20‰—*F*_3,20_ = 6.23, *P* < 0.01). Although salinity acclimation did not alter RBC pH at rest, differences were present at 0.5 h post-SAS (*F*_2,16_ = 14.65, *P* < 0.001), with a *post hoc* test indicating the 10 and 20‰ groups were not different from each other, but were both lower than the 0‰ group. RBC pH largely recovered by 8 h post-SAS but significant differences in pH remained (*F*_2,16_ = 4.29, *P* < 0.05); *post hoc* test indicated this was due to differences between the 0 and 20‰ groups. There was a significant effect of time postexercise (*F*_3,62_ = 3.10, *P* < 0.05) and salinity acclimation (*F*_2,62_ = 3.81, *P* < 0.05) on heart pH, with *post hoc* analysis indicating that heart pH_i_ in the 10‰ group was significantly lower than those in the FW group at 0.5 h post-SAS (Fig. 2C).

### Secondary stress indicators

**Figure 4:**
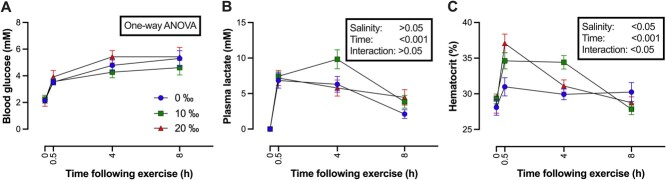
The effect of manual chasing on (A) blood glucose, (B) plasma lactate and (C) hematocrit in 3-year-old WS (*Acipenser transmontanus*) acclimated to 0 (blue lines), 10 (green lines) or 20‰ (red lines) and sampled at rest (0 h), 0.5, 4 or 8 h. Errors bars represent SEM. See text for results of one-way ANOVA.

Salinity treatment did not affect blood glucose concentrations, either before or after the angling challenge. Changes in blood glucose did occur after SAS at each salinity level (0‰—H = 17.62 (2), *P* < 0.001; 10‰—*F*_3,21_ = 6.288, *P* < 0.01; 20‰—*F*_3,21_ = 7.385, *P* < 0.01) and resulted in roughly a doubling of glucose levels at 0.5 h postexercise, with *post hoc* analysis indicating a significant increase in blood glucose at 4 and 8 h in all salinities compared with controls (Fig. 4A).

As with glucose, plasma lactate levels were also not affected by salinity treatment in WS at rest (*F*_2,65_ = 2.382, *P* = 0.10); however, time after exercise significant (*F*_3,64_ = 36.67, *P* < 0.0001; Fig. 4B). A *post hoc* test indicated that lactate levels increased at 0.5 h post-SAS compared with rest in all salinities. In addition, the *post hoc* confirmed that at 4 h post-SAS, lactate levels were higher in the 10‰ group compared with the 0 and 20‰ groups.

Hct was not affected by salinity acclimation (*F*_2,13_ = 0.3893, *P* = 0.68). At 0.5 and 4 h post-SAS, Hct differed between salinities (0.5 h: *F*_2,16_ = 6.204, *P* < 0.01; 4 h: *F*_2,18_ = 7.375, *P* < 0.01) but no differences were detected at 8 h post-SAS (*F*_2,17_ = 1.470, *P* = 0.26). In the 10 and 20‰ groups, Hct differed post-SAS (10‰—*F*_3,22_ = 15.19, *P* < 0.0001; 20‰—*F*_3,21_ = 14.13, *P* < 0.001) (Fig. 4C).

## Discussion

### Three-year old white sturgeon acclimated to 10 and 20‰

WS aged 3 y were able to acclimate to elevated salinity of 10 or 20‰ through a 2-wk period. No significant differences were observed in plasma osmolarity among groups after salinity treatment (Fig. 1A), and these values were similar to those reported by other studies measuring osmolarity in WS (McEnroe and Cech, 1985; Tashjian *et al.,* 2007; Amiri *et al.,* 2009; Shaughnessy *et al.,* 2015), although less than those of juvenile Siberian sturgeon (*A. baerii*), Chinese sturgeon (*A. sinensis*), and Persian sturgeon (*A. persicus*) acclimated to 14‰ (Rodríguez *et al.,* 2002), 10‰ (He *et al.,* 2008) or 11‰ (Shirangi *et al.,* 2016), respectively. The small but insignificant increase in plasma osmolarity between FW and 10 and 20‰ is likely due in part to the change in plasma [Cl^−^], which increased from 104.5 mM in FW to and 113.4 and 120.4 mM in 10 and 20‰, respectively (Fig. 1B). Despite these increased levels, values for osmolarity and plasma [Cl^−^] in WS were slightly lower than the values for a typical euryhaline teleost (Edwards and Marshall, 2013); this has been previously noted (Shaughnessy *et al.,* 2015).

As an anadromous species, WS spawn and spend the first part of their life in FW, and at some point, they become capable of entering seawater. Unlike salmonids, another group of anadromous fishes, smoltification in sturgeon is not characterized by known morphological, behavioural and physiological changes; however, changes in hormones (e.g. growth hormone) and Na^+^, K^+^-ATPase do occur (Hasegawa *et al.,* 1987; Rodríguez *et al.,* 2002; Allen *et al.,* 2011). Anadromous sturgeon, including WS, will enter seawater after a couple of years of FW residence (Zydlewski and Wilkie, 2013); the exact timing of seawater entry, although not clear, is believed to be dictated by a combination of age and size. Evidence for this is in the finding that larger juvenile WS experience lower mortality when transferred to elevated salinity (Zydlewski and Wilkie, 2013). In addition, McEnroe and Cech (1985) found that in the 0.4- to 0.6-g size class, there was only 50% survival after transfer to 10‰ and <5% survival after transfer to 15‰; not until fish reached 4.9—9.5 g did survival reach 100% after transfer to both salinities; similar findings were observed by others (Amiri *et al.,* 2009) and also in gulf sturgeon (*A. oxyrhynchus desotoi*) (Altinok *et al.,* 1998). Interestingly, WS do not seem to have a “smoltification window” beyond which salinity acclimation is not possible, as occurs in some salmonids (pink and chum salmon) (Hasegawa *et al.,* 1987).

Salinity acclimation, as indicated by seawater entry, varies amongst anadromous species. Together, the aforementioned findings indicate that although sturgeon do have some capacity for salinity tolerance at early life stages, they are not capable of surviving long-term in hyperosmotic full-strength seawater at less than 30 g (Amiri *et al.,* 2009). Previous exposure to brackish water does seem to make the transition from FW to seawater (SW) less challenging though (McKenzie *et al.,* 2001). The closely related green sturgeon spend long portions of their life history in marine waters and brackish estuaries (Lindley *et al.,* 2008), and unsurprisingly seem capable of earlier seawater entry than other sturgeon species because they reached 100% survival to 20‰ at 60 d posthatch (<5 grams) and 100% survival to 30 and 34‰ at 100 and 135 d posthatch, respectively (Allen and Cech, 2007; Lindley *et al.,* 2008; Hildebrand *et al.,* 2016). Sea lamprey (*Petromyzon marinus*) have an FW phase (ammocoete) that typically lasts for 3–7 y followed by the parasitic phase at sea (McCormick, 2013; Zydlewski and Wilkie, 2013). Another basal fish species, alligator gar (*Atractosteus spatula*), can survive as a juvenile (<1 y, 185 g) in salinities <24‰ for >30 d, but longer term survival depends on returning to lower salinities (Schwarz and Allen, 2014). Differences exist between the closely related Pacific salmonids, where pink (*Oncorhynchus gorbuscha*) and chum (*O. keta*) enter seawater soon after hatch, whereas others such as coho (*O. kisutch*) and steelhead trout (*O. mykiss*) may spend one to several years in FW (McCormick, 2013). WS, in contrast to those aforementioned examples, tend to remain in FW or brackish environments, with limited marine excursions (Hildebrand *et al.,* 2016). This migratory pattern of returning to FW to recover from saline excursions may be similar for small WS, and may also explain why more easily identified smoltification characteristics are less present.

### Metabolic acidosis after exercise

Salinity acclimation did not alter resting blood pH, nor did the severity of acid–base disturbance in the blood after SAS differ among salinity treatments. Blood pH was reduced by an average of 0.26 pH units 0.5 h after SAS (Fig. 2A). Exercise-induced metabolic acidosis is a common response observed amongst fishes (Wood, 1991; Milligan, 1996; Nelson *et al.,* 1996; Harter *et al.,* 2014), including WS (Shartau *et al.,* 2017). The changes in pH_e_ reported here are similar to those reported by Shartau *et al*. (2017), who observed a reduction in pH_e_ from 7.7 to 7.4 0.25 h post-SAS in WS in FW.

RBC pH_i_ after acclimation to 20‰ was significantly lower than either FW or 10‰ (*P* < 0.05). At 0.5 h after SAS, RBC pH_i_ was reduced in 10‰, 4 h in 0‰ (P < 0.05), which recovered by 8 h. In the 20‰ group, RBC pH_i_ did not significantly change; this may be a result of the lower initial starting pH_i_. RBC pH_i_ in resting FW fish in this study was 7.56 pH units, which was higher than the previously reported value of 7.2 pH units by Shartau *et al*. (2017) and 7.35 pH units by Baker *et al*. (2009). Interestingly, Shartau *et al*. (2017) observed a reduction in RBC pH_i_ 0.25 h postexercise in FW sturgeon exercised to exhaustion using a similar methodology—here, no significant reduction was observed. This odd difference suggests that either the pH_i_ reduction was not captured by the time points sampled here (and may be compensated for exceedingly quickly) or the severity of the exercise was perhaps less.

Heart pH_i_ remained unchanged after SAS at all salinities; however, there were insignificant reductions occurring within the first 0.5 h (Fig. 2C), which is similar to what was observed by Shartau *et al*. (2017), who reported a small but significant reduction in heart pH 2 h postexercise in 0‰ fish. The reduction seems to be the most pronounced in the 10‰, and although it is not statistically significant, this may represent a change in capacity for heart pH_i_ regulation. Salinity acclimation is associated with altered gene expression: gill tissue of Siberian sturgeon (Guo *et al.,* 2018), medaka (*Oryzias melastigma*) (Liang *et al.,* 2021), stickleback (*Gasterosteus aculeatus*) (Gibbons *et al.,* 2017), and *Argyrosomus japonicus* (Li *et al.,* 2021) all exhibited differential expression of genes associated with ion transport and cell permeability to ions and water. Medaka also exhibited changes in differential expression of genes in liver associated with protein synthesis and metabolism (Liang *et al.,* 2021). Given the changes observed in other species, it is likely WS also experience changes in gene and protein expression after salinity. This exposure may alter how tissues, such as the heart, regulate ions, including those associated with pH regulation, resulting in biologically relevant impacts on heart function given that small pH changes may have consequences for transporter function and activity (Parks *et al.,* 2008). Of particular interest for further study may be that, in WS, an isoosmotic environment may induce expression of genes that are substantially different than those induced by a hyperosmotic one. The timing of the exposure could very well also play an important role in this migratory fish.

### Impact of salinity acclimation on recovery from SAS

Acclimation to salinities similar to those found in the Fraser River and Fraser River estuary did not alter the capacity of 3-y-old WS to recover from SAS because the severity of acidosis and time course of recovery were similar between salinity treatments. Even so, differences in plasma [Cl^−^], [HCO_3_^−^], [*P*CO_2_], and lactate concentrations suggest that salinity acclimation may alter the mechanisms involved in acid–base homeostasis associated with exercise-induced metabolic acidosis, which may have consequences for regulation of tissue pH.

**Figure 5:**
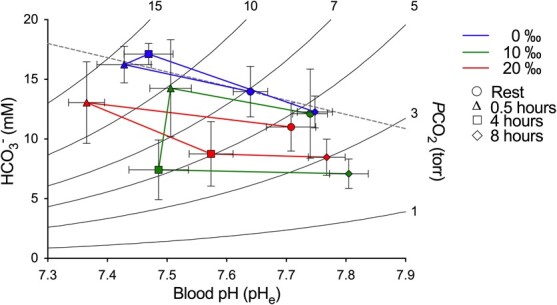
Changes in blood pH and HCO_3_^−^ after SAS in 3-y-old WS (*Acipenser transmontanus*) acclimated to different salinities. Changes in blood pH (pHe) and plasma [HCO_3_^−^] are presented on a pH-HCO_3_^−^ plot (Davenport diagram); curved lines indicated *P*CO_2_ isopleths. Fish were acclimated in either 0 (blue lines), 10 (green lines) or 20‰ (red lines) and sampled at rest ([INSERT FX]), 0.5 h ([INSERT FX]), 4 h ([INSERT FX]) or 8 h ([INSERT FX]) after stimulated angling stress.

Changes in recovery pattern between salinities (Fig. 5) indicate that at 4 h postexercise, fish in 10 and 20‰ exhibited a metabolic acidosis, whereas fish in 0‰ did not. Because only sufficient numbers of sturgeon were available to examine four time points, this may not have allowed full capture of all changes, as previously, Shartau *et al*. (2017) found that WS in FW experienced a metabolic acidosis within 0.25 h postexercise, and this persisted for at least 2 h. That there was no metabolic acidosis apparent in FW fish in this study suggests that either recovery happened more rapidly in the current study or that the severity of the exercise was less than in Shartau *et al*. (2017).

The modest differences between the responses of WS to SAS in differing salinities are likely associated with changes to transporter isoform expression and activity in tissues during salinity change. In particular, acclimation to increased salinity induces significant changes in transporter isoforms and activity at the gills. In other sturgeon species, transfer from FW to brackish water induces an increase in Na+-K+-ATPase (NKA) activity (Rodríguez *et al.,* 2002; He *et al.,* 2008; Shirangi *et al.,* 2016); this was also observed in other euryhaline fishes (Richards *et al.,* 2003; Scott *et al.,* 2004; Evans *et al.,* 2005; Nilsen *et al.,* 2007; Urbina *et al.,* 2013; Esbaugh *et al.,* 2019). These changes in NKA activity and expression can also occur in the kidneys (Marshall and Grosell, 2005; Yang *et al.,* 2016) and intestines (Fuentes *et al.,* 1997; Schwarz and Allen, 2014). In green sturgeon acclimated to high salinity, upregulation of NKCC (Na+, K+, 2Cl− transporter) and downregulation of V-type H + -ATPase (VHA) occurred in the gills (Sardella and Kültz, 2009). In Atlantic salmon gills, changes to NKCC and cystic fibrosis transmembrane conductance regulator (CFTR) have been observed after acclimation to higher salinity (Nilsen *et al.,* 2007). Certainly, the possibility that similar changes in WS could drive differences in the capacity or timing of the organismal response to SAS is worth examining, especially in light of potential mortality of an endangered species impacted by a C&R fishery.

Many fishes including WS are able to preferentially regulate pH_i_, which provides tremendous capacity for pH_i_ regulation during acid–base disturbances; however, this strategy has only been examined in FW-acclimated fish (Shartau *et al.,* 2016, 2019, 2020). The changes in ion transporters at the tissue level described previously may subsequently affect pH_i_ regulation, which in sturgeon is likely one of the most protective responses to an acid–base disturbance. The capacity for pH_i_ regulation of RBC and heart seems to be reduced in higher salinities, which may suggest changes to transporter isoforms and activity when responding to higher salinities has consequences for pH_i_ regulation. Regulation of pH_i_ has been examined after SAS and fishes using preferential pH_i_ regulation seem to be largely capable of protecting pH_i_ after exercise (Harter *et al.,* 2014; Shartau *et al.,* 2017), although this may represent a greater challenge than respiratory acidosis in WS, perhaps due to high lactate concentration (this study; Shartau *et al.,* 2017). However, should pH_i_ regulation be more difficult in WS acclimated to higher salinities, the potential increase in recovery time may expose large WS to greater predation or higher incidental mortality. Thus, whether mechanisms of preferential pH_i_ regulation yet to be described are less effective at high salinity is a topic that should be investigated.

### Concluding points

Acclimation within 2 w of 3-y-old WS to salinities up to 20‰ does not pose a challenge, and once acclimation is achieved, it does not seem to greatly change their response to SAS. Because WS support economically important C&R fisheries in British Columbia (Glova *et al.,* 2009), knowing how they recover from SAS informs on their ability to recover from C&R (McLean *et al.,* 2016, 2019, 2020). Due to the salt wedge that extends up the Fraser River (Murray, 2016; Leung *et al.,* 2018), WS may be acclimated to different salinities within the region where C&R fisheries occurs. Based on these results, there are not likely any physiological challenges facing WS after the SAS if they are caught in the different salinity regions of the Fraser River. In addition, because sturgeon of the size and age examined here were capable of acclimation, future studies aimed at determining when between the size of 30 g (Amiri *et al.,* 2009) and 3-y-old, 3-kg WS (this study) become salinity tolerant would provide further insight into the migratory patterns of young WS. It may be important for conservation managers and researchers to consider how other environmental factors alter recovery from SAS. This may include changes in temperature and dissolved oxygen because these are known to impact fish recovery from exercise (Randall and Brauner, 1991; Deslauriers and Kieffer, 2012; Mandal *et al.,* 2016), and upstream regions of the Fraser River are likely to experience greater variation in these environmental parameters compared with those further downstream nearest the ocean.

Even though the risk of C&R mortality seems to be unaffected by salinity acclimation, a further risk might be associated with the saline waters in the Fraser. Should an FW- or SW-acclimated fish be caught and then returned into water of different salinity, risk factors for mortality may be higher, particularly if recovery is slower than normal. As this study suggests, salinity acclimation alters the pattern by which recovery from the metabolic acidosis occurs, it warrants further investigation of various scenarios that could happen in fishing areas closer to the tidal boundaries for this fishery.

## Funding

This work was supported by Natural Sciences and Engineering Research Council (NSERC) of Canada Discovery Grant (RGPIN-2017-06895) to D.W.B.

## Conflicts of Interest Statement

The authors have no conflicts to declare.

## Data Availability

Datasets are available on request: The raw data supporting the conclusions of this article will be made available by the authors, without undue reservation.

## Author Contributions

Conceptualization: R.B.S., D.W.B.; Methodology: R.B.S., D.W.B.; Investigation: R.B.S., J.S., D.W.B.; Formal analysis: R.B.S., D.W.B.; Writing original draft: R.B.S., D.W.B.; Writing—reviewing and editing: R.B.S., J.S., D.W.B.; Project administration: D.W.B.; Funding acquisition: D.W.B.

## References

[ref1] Allen PJ , Cech JJ (2007) Age/size effects on juvenile green sturgeon, *Acipenser medirostris*, oxygen consumption, growth, and osmoregulation in saline environments. Environ Biol Fishes 79: 211–229. 10.1007/s10641-006-9049-9.

[ref2] Allen PJ , McEnroe M, Forostyan T, Cole S, Nicholl MM, Hodge B, Cech JJ (2011) Ontogeny of salinity tolerance and evidence for seawater-entry preparation in juvenile green sturgeon, *Acipenser medirostris*. J Comp Physiol B 181: 1045–1062. 10.1007/s00360-011-0592-0. 21630040

[ref3] Allen PJ , Mitchell ZA, DeVries RJ, Aboagye DL, Ciaramella MA, Ramee SW, Stewart HA, Shartau RB (2014) Salinity effects on Atlantic sturgeon (*Acipenser oxyrinchus oxyrinchus* Mitchill, 1815) growth and osmoregulation. J Appl Ichthyol 30: 1229–1236. 10.1111/jai.12542.

[ref4] Altinok I , Galli SM, Chapman FA (1998) Ionic and osmotic regulation capabilities of juvenile Gulf of Mexico sturgeon, *Acipenser oxyrinchus de sotoi*. Comp Biochem Physiol A 120: 609–616. 10.1016/S1095-6433(98)10073-9.

[ref5] Amiri BM , Baker DW, Morgan JD, Brauner CJ (2009) Size dependent early salinity tolerance in two sizes of juvenile white sturgeon, *Acipenser transmontanus*. Aquaculture 286: 121–126. 10.1016/j.aquaculture.2008.08.037.

[ref6] Baker DW , May C, Brauner CJ (2009) A validation of intracellular pH measurements in fish exposed to hypercarbia: the effect of duration of tissue storage and efficacy of the metabolic inhibitor tissue homogenate method. J Fish Biol 75: 268–275. 10.1111/j.1095-8649.2009.02261.x. 20738495

[ref7] Baker DW , Wood AM, Kieffer JD (2005a) Juvenile Atlantic and Shortnose sturgeons (family: Acipenseridae) have different hematological responses to acute environmental hypoxia. Physiol Biochem Zool 78: 916–925. 10.1086/432860. 16228931

[ref8] Baker DW , Wood AM, Litvak MK, Kieffer JD (2005b) Haematology of juvenile *Acipenser oxyrinchus* and *Acipenser brevirostrum* at rest and following forced activity. J Fish Biol 66: 208–221. 10.1111/j.0022-1112.2005.00595.x.

[ref8a] Boutilier RG , Heming TA, Iwama GK (1984) Appendix: physicochemicalparameters for use in fish respiratory physiology. In WS Hoar, DJ Randall, eds, Fish physiology. Fish physiology. Academic, New York pp 403–430

[ref8b] Brauner CJ , Wang T, Wang Y (2004) Limited extracellular butcomplete intracellular acid-base regulation during short-termenvironmental hypercapnia in the armoured catfish, Liposarcuspardalis J Exp Biol 207: 3381–3390. 10.1242/jeb.01144. 15326214

[ref9] Brown ABJ , Kieffer JD (2019) Does body size affect the response to exercise in shortnose sturgeon (*Acipenser brevirostrum*)? J Appl Ichthyol 35: 69–77. 10.1111/jai.13743.

[ref10] Byrne JM , Beamish FWH, Saunders RL (1972) Influence of salinity, temperature, and exercise on plasma osmolality and ionic concentration in Atlantic salmon (*Salmo salar*). J Fish Res Board Can 29: 1217–1220. 10.1139/f72-181.

[ref11] Christensen EAF , Illing B, Iversen NS, Johansen JL, Domenici P, Steffensen JF (2018) Effects of salinity on swimming performance and oxygen consumption rate of shiner perch *Cymatogaster aggregata*. J Exp Mar Biol Ecol 504: 32–37. 10.1016/j.jembe.2018.04.002.

[ref11a] City of Nanaimo (2015) Water quality report. See http://www.nanaimo.ca/assets/Departments/Engineering∼Public∼Works/Water∼Supply/Publications∼and∼Forms/AnnualWaterQualityReport2015.pdf.

[ref12] Conte F , Doroshov S, Lutes P, Strange E (1988) Hatchery Manual for the White Sturgeon (Acipenser Transmontanus Richardson) with Application to Other North American Acipenseridae. Division of Agricultural and Natural Resources, University of California, Oakland, CA

[ref13] COSEWIC (2004) COSEWIC assessment and update status report on the green sturgeon *Acipenser medirostris* in Canada. In Committee on the Status of Endangered Wildlife in Canada Ottawa. vii + 31 pp.

[ref14] Damsgaard C , McGrath M, Wood CM, Richards JG, Brauner CJ (2020) Ion-regulation, acid/base-balance, kidney function, and effects of hypoxia in coho salmon, *Oncorhynchus kisutch*, after long-term acclimation to different salinities. Aquaculture 528: 735571. 10.1016/j.aquaculture.2020.735571.

[ref15] Deslauriers D , Kieffer JD (2012) The effects of temperature on swimming performance of juvenile shortnose sturgeon (*Acipenser brevirostrum*). J Appl Ichthyol 28: 176–181. 10.1111/j.1439-0426.2012.01932.x.

[ref16] Edwards SL , Marshall WS (2013) Principles and patterns of osmoregulation and euryhalinity in fishes. In SD McCormick, AP Farrell, CJ Brauner, eds, Fish Physiology (Euryhaline Fishes), Ed32nd. Cambridge, CA: Academic Press, pp. 1–44 10.1016/B978-0-12-396951-4.00001-3

[ref17] Esbaugh AJ , Brix KV, Grosell M (2019) Na^+^ K^+^ ATPase isoform switching in zebrafish during transition to dilute freshwater habitats. Proc R Soc B 286: 20190630. 10.1098/rspb.2019.0630. PMC6545080 31113326

[ref18] Evans DH , Piermarini PM, Choe KP (2005) The multifunctional fish gill: dominant site of gas exchange, osmoregulation, acid-base regulation, and excretion of nitrogenous waste. Physiol Rev 85: 97–177. 10.1152/physrev.00050.2003. 15618479

[ref19] Faught E , Aluru N, Vijayan MM (2016) Biology of stress in fishes. In CB Schreck, L Tort, AP Farrell, CJ Brauner, eds, Fish Physiology. (London: Academic Press), pp. 113–166

[ref20] Faught E , Vijayan MM (2018) Maternal stress and fish reproduction: the role of cortisol revisited. Fish Fish 19: 1016–1030. 10.1111/faf.12309.

[ref21] Ferguson RA , Tufts BL (1992) Physiological effects of brief air exposure in exhaustively exercised rainbow trout (*Oncorhynchus mykiss*): implications for “catch and release” fisheries. Can J Fish Aquat Sci 49: 1157–1162. 10.1139/f92-129.

[ref22] Fuentes J , Soengas JL, Rey P, Rebolledo E (1997) Progressive transfer to seawater enhances intestinal and branchial Na^+^-K^+^-ATPase activity in non-anadromous rainbow trout. Aquacult Int 5: 217–227. 10.1023/A:1018387317893.

[ref23] Gibbons TC , Metzger DCH, Healy TM, Schulte PM (2017) Gene expression plasticity in response to salinity acclimation in threespine stickleback ecotypes from different salinity habitats. Mol Ecol 26: 2711–2725. 10.1111/mec.14065. 28214359

[ref24] Glova G , Nelson T, Roberts R (2009) A White Sturgeon Habitat Conservation and Protection Strategy for Lower Fraser River 2008-09. Fraser River Sturgeon Conservation Society. Vancouver, BC.

[ref25] Guo B , Tang Z, Wu C, Xu K, Qi P (2018) Transcriptomic analysis reveal an efficient osmoregulatory system in Siberian sturgeon *Acipenser baeri* in response to salinity stress. Sci Rep 8: 14353. 10.1038/s41598-018-32771-x. 30254302 PMC6156415

[ref26] Harter TS , Shartau RB, Baker DW, Jackson DC, Val AL, Brauner CJ (2014) Preferential intracellular pH regulation represents a general pattern of pH homeostasis during acid–base disturbances in the armoured catfish, *Pterygoplichthys pardalis*. J Comp Physiol B 184: 709–718. 10.1007/s00360-014-0838-8. 24973965

[ref27] Hasegawa S , Hirano T, Ogasawara T, Iwata M, Akiyama T, Arai S (1987) Osmoregulatory ability of chum salmon, *Oncorhynchus keta*, reared in fresh water for prolonged periods. Fish Physiol Biochem 4: 101–110. 10.1007/BF02044319. 24226149

[ref28] He X , Zhuang P, Zhang L, Xie C (2008) Osmoregulation in juvenile Chinese sturgeon (*Acipenser sinensis* gray) during brackish water adaptation. Fish Physiol Biochem 35: 223–230. 10.1007/s10695-008-9230-5. 19343518

[ref29] Hildebrand LR , Schreier AD, Lepla K, McAdam SO, McLellan J, Parsley MJ, Paragamian VL, Young SP (2016) Status of white sturgeon (*Acipenser transmontanus* Richardson, 1863) throughout the species range, threats to survival, and prognosis for the future. J Appl Ichthyol 32: 261–312. 10.1111/jai.13243.

[ref30] Kieffer JD , May LE (2020) Repeat UCrit and endurance swimming in juvenile shortnose sturgeon (*Acipenser brevirostrum*). J Fish Biol 96: 1379–1387. 10.1111/jfb.14306. 32128813

[ref31] Kieffer JD , Wakefield AM, Litvak MK (2001) Juvenile sturgeon exhibit reduced physiological responses to exercise. J Exp Biol 204: 4281–4289. 10.1242/jeb.204.24.4281. 11815652

[ref32] Larsen BK , Jensen FB (1997) Influence of ionic composition on acid-base regulation in rainbow trout (*Oncorhynchus mykiss*) exposed to environmental hypercapnia. Fish Physiol Biochem 16: 157–170. 10.1007/BF00004672.

[ref33] Leung ATY , Stronach J, Matthieu J (2018) Modelling behaviour of the salt wedge in the Fraser River and its relationship with climate and man-made changes. J Mar Sci Eng 6: 130. 10.3390/jmse6040130.

[ref34] Li Z , Gao T, Han Z (2021) RNA-seq and analysis of *Argyrosomus japonicus* under different salinities. Front Mar Sci 8: 790065. 10.3389/fmars.2021.790065.

[ref35] Liang P , Saqib HSA, Lin Z, Zheng R, Qiu Y, Xie Y, Ma D, Shen Y (2021) RNA-seq analyses of marine Medaka (*Oryzias melastigma*) reveals salinity responsive transcriptomes in the gills and livers. Aquat Toxicol 240: 105970. 10.1016/j.aquatox.2021.105970. 34562875

[ref36] Lindley ST , Moser ML, Erickson DL, Belchik M, Welch DW, Rechisky EL, Kelly JT, Heublein J, Klimley AP (2008) Marine migration of North American green sturgeon. T Am Fish Soc 137: 182–194. 10.1577/T07-055.1.

[ref37] Mandal P , Cai L, Tu Z, Johnson D, Huang Y (2016) Effects of acute temperature change on the metabolism and swimming ability of juvenile sterlet sturgeon (*Acipenser ruthenus*, Linnaeus 1758). J Appl Ichthyol 32: 267–271. 10.1111/jai.13033.

[ref38] Marshall WS , Grosell M (2005) Ion transport, osmoregulation, and acid-base balance. In The Physiology of Fishes (3rd ed.), edited by D Evans, JB Claiborne. Baco Raton, FL: CRC, pp. 177–230

[ref39] McCormick SD (2013) Smolt Physiology and Endocrinology. In SD McCormick, AP Farrell, CJ Brauner, eds, Fish Physiology - Euryhaline Fishes. (Cambridge, CA: Academic Press), pp. 199–251

[ref40] McEnroe M , Cech J (1985) Osmoregulation in juvenile and adult white sturgeon, *Acipenser transmontanus*. Environ Biol Fishes 14: 23–30. 10.1007/BF00001573.

[ref41] McKenzie DJ , Cataldi E, Romano P, Taylor EW, Cataudella S, Bronzi P (2001) Effects of acclimation to brackish water on tolerance of salinity challenge by young-of-the-year Adriatic sturgeon (*Acipenser naccarii*). Can J Fish Aquat Sci 58: 1113–1121. 10.1139/f01-058.

[ref42] McLean MF , Hanson KC, Cooke SJ, Hinch SG, Patterson DA, Nettles TL, Litvak MK, Crossin GT (2016) Physiological stress response, reflex impairment and delayed mortality of white sturgeon *Acipenser transmontanus* exposed to simulated fisheries stressors. Conserv Physiol 4: cow031. 10.1093/conphys/cow031. 27766153 PMC5070429

[ref43] McLean MF , Litvak MK, Cooke SJ, Hanson KC, Patterson DA, Hinch SG, Crossin GT (2019) Immediate physiological and behavioural response from catch-and-release of wild white sturgeon (*Acipenser transmontanus* Richardson, 1836). Fish Res 214: 65–75. 10.1016/j.fishres.2019.02.002.

[ref44] McLean MF , Litvak MK, Stoddard EM, Cooke SJ, Patterson DA, Hinch SG, Welch DW, Crossin GT (2020) Linking environmental factors with reflex action mortality predictors, physiological stress, and post-release movement behaviour to evaluate the response of white sturgeon (*Acipenser transmontanus* Richardson, 1836) to catch-and-release angling. Comp Biochem Physiol A 240: 110618. 10.1016/j.cbpa.2019.110618. 31726105

[ref45] Meade JW (1985) Allowable ammonia for fish culture. Prog Fish Cult 47: 135–145. 10.1577/1548-8640(1985)47<135:AAFFC>2.0.CO;2.

[ref46] Milligan CL (1996) Metabolic recovery from exhaustive exercise in rainbow trout. Comp Biochem Physiol A 113: 51–60. 10.1016/0300-9629(95)02060-8.

[ref47] Murray A (2018) Fraser River Delta: Southern British Columbia (Canada) In The Wetland Book; CM Finlayson, GR Milton, RC Prentice, NC Davidson, Eds.; Springer: Dordrecht, The Netherlands, pp. 565–575.

[ref48] Nelson J , Tang Y, Boutilier R (1996) The effects of salinity change on the exercise performance of two Atlantic cod (*Gadus morhua*) populations inhabiting different environments. J Exp Biol 199: 1295–1309. 10.1242/jeb.199.6.1295. 9319167

[ref49] Nelson T , Robichaud D, Challenger W, English K, Mochizuki T, Thibault T, Rissling J, Gazey W (2019) Lower Fraser River White Sturgeon Monitoring and Assessment Program. In Status of White Sturgeon in the Lower Fraser River in 2018 With Abundance Estimates Derived from 24-Month Bayesian Mark Recapture Modeling. Fraser River Sturgeon Conservation Society, Vancouver, BC.

[ref50] Nelson TC , Gazey WJ, English KK, Rosenau ML (2013) Status of white sturgeon in the Lower Fraser River, British Columbia. Fisheries 38: 197–209. 10.1080/03632415.2013.777664.

[ref51] Nilsen TO , Ebbesson LOE, Madsen SS, McCormick SD, Andersson E, Björnsson BT, Prunet P, Stefansson SO (2007) Differential expression of gill Na^+^,K^+^-ATPaseα- and β-subunits, Na^+^,K^+^,2Cl^−^ cotransporter and CFTR anion channel in juvenile anadromous and landlocked Atlantic salmon *Salmo salar*. J Exp Biol 210: 2885–2896. 10.1242/jeb.002873. 17690237

[ref52] Pankhurst NW , Dedualj M (1994) Effects of capture and recovery on plasma levels of cortisol, lactate and gonadal steroids in a natural population of rainbow trout. J Fish Biol 45: 1013–1025. 10.1111/j.1095-8649.1994.tb01069.x.

[ref53] Parks SK , Tresguerres M, Goss GG (2008) Theoretical considerations underlying Na^+^ uptake mechanisms in freshwater fishes. Comp Biochem Physiol C 148: 411–418. 10.1016/j.cbpc.2008.03.002. 18420463

[ref54] Pedersen LF , Malte H (2004) Repetitive acceleration swimming performance of brown trout in fresh water and after acute seawater exposure. J Fish Biol 64: 273–278. 10.1111/j.1095-8649.2004.00288.x.

[ref55] Penny FM , Kieffer JD (2019) Lack of change in swimming capacity (Ucrit) following acute salinity exposure in juvenile shortnose sturgeon (*Acipenser brevirostrum*). Fish Physiol Biochem 45: 1167–1175. 10.1007/s10695-019-00629-2. 30874954

[ref56] Pörtner H , Boutilier R, Tang Y, Toews D (1991) Determination of intracellular pH and PCO_2_ after metabolic inhibition by fluoride and nitrilotriacetic acid. Resp Physiol 81: 255–273. 10.1016/0034-5687(90)90050-9. 2124717

[ref57] Randall D , Brauner C (1991) Effects of environmental factors on exercise in fish. J Exp Biol 160: 113–126. 10.1242/jeb.160.1.113.

[ref58] Redding JM , Schreck CB (1983) Influence of ambient salinity on osmoregulation and cortisol concentration in yearling Coho salmon during stress. T Am Fish Soc 112: 800–807. 10.1577/1548-8659(1983)112<800:IOASOO>2.0.CO;2.

[ref59] Richards JG , Semple JW, Bystriansky JS, Schulte PM (2003) Na^+^/K^+^-ATPase-isoform switching in gills of rainbow trout (*Oncorhynchus mykiss*) during salinity transfer. J Exp Biol 206: 4475–4486. 10.1242/jeb.00701. 14610032

[ref60] Robichaud D , English KK, Bocking RC, Nelson TC (2006) Direct and Delayed Mortality of White Sturgeon Caught in Three Gear-Types in the Lower Fraser River. Report prepared for Tsawwassen First Nation Fisheries, Delta, BC, LGL Limited, Sidney, BC.

[ref61] Rodríguez A , Gallardo MA, Gisbert E, Santilari S, Ibarz A, Sánchez J, Castelló-Orvay F (2002) Osmoregulation in juvenile Siberian sturgeon (*Acipenser baerii*). Fish Physiol Biochem 26: 345–354. 10.1023/B:FISH.0000009263.83075.68.

[ref62] Sackville MA , Shartau RB, Damsgaard C, Hvas M, Phuong LM, Wang T, Bayley M, Huong DTT, Phuong NT, Brauner CJ (2018) Water pH limits extracellular but not intracellular pH compensation in the CO_2_-tolerant freshwater fish *Pangasianodon hypophthalmus*. J Exp Biol 221: jeb190413-5. 10.1242/jeb.190413. 30352827

[ref63] Sardella B , Kültz D (2009) Osmo- and ionoregulatory responses of green sturgeon (*Acipenser medirostris*) to salinity acclimation. J Comp Physiol B 179: 383–390. 10.1007/s00360-008-0321-5. 19066909

[ref64] Schwarz DE , Allen PJ (2014) Effects of salinity on growth and ion regulation of juvenile alligator gar *Atractosteus spatula*. Comp Biochem Physiol A 169: 44–50. 10.1016/j.cbpa.2013.12.012. 24368134

[ref65] Scott GR , Richards JG, Forbush B, Isenring P, Schulte PM (2004) Changes in gene expression in gills of the euryhaline killifish *Fundulus heteroclitus* after abrupt salinity transfer. Am J Physiol Cell Physiol 287: C300–C309. 10.1152/ajpcell.00054.2004. 15044150

[ref66] Shartau RB , Baker DW, Brauner CJ (2017) White sturgeon (*Acipenser transmontanus*) acid–base regulation differs in response to different types of acidoses. J Comp Physiol B 187: 985–994. 10.1007/s00360-017-1065-x. 28283796

[ref67] Shartau RB , Baker DW, Crossley DA, Brauner CJ (2016) Preferential intracellular pH regulation: hypotheses and perspectives. J Exp Biol 219: 2235–2244. 10.1242/jeb.126631. 27489212

[ref68] Shartau RB , Baker DW, Harter TS, Aboagye DL, Allen PJ, Val AL, Crossley DA, Kohl ZF, Hedrick MS, Damsgaard C et al. (2020) Preferential intracellular pH regulation is a common trait amongst fishes exposed to high environmental CO_2_. J Exp Biol 223: jeb.208868. 10.1242/jeb.208868. 32127382

[ref69] Shartau RB , Damsgaard C, Brauner CJ (2019) Limits and patterns of acid-base regulation during elevated environmental CO_2_ in fish. Comp Biol Phys A 236: 110524. 10.1016/j.cbpa.2019.110524. 31301422

[ref70] Shaughnessy CA , Baker DW, Brauner CJ, Morgan JD, Bystriansky JS (2015) Interaction of osmoregulatory and acid-base compensation in white sturgeon (*Acipenser transmontanus*) during exposure to aquatic hypercarbia and elevated salinity. J Exp Biol 218: 2712–2719. 10.1242/jeb.125567. 26333926

[ref71] Shedko SV (2022) Molecular dating of phylogeny of sturgeons (Acipenseridae) based on total evidence analysis. Russ J Genet+ 58: 718–729. 10.1134/S1022795422060084.

[ref72] Shirangi SA , Kalbassi MR, Khodabandeh S, Jafarian H, Lorin-Nebel C, Farcy E, Lignot J-H (2016) Salinity effects on osmoregulation and gill morphology in juvenile Persian sturgeon (*Acipenser persicus*). Fish Physiol Biochem 42: 1741–1754. 10.1007/s10695-016-0254-y. 27341821

[ref73] Tashjian D , Cech JJ, Hung SSO (2007) Influence of dietary l-selenomethionine exposure on the survival and osmoregulatory capacity of white sturgeon in fresh and brackish water. Fish Physiol Biochem 33: 109. 10.1007/s10695-006-9122-5.

[ref74] Tovey KJ , Brauner CJ (2017) Effects of water ionic composition on acid–base regulation in rainbow trout, during hypercarbia at rest and during sustained exercise. J Comp Physiol B 188: 295–304. 29067494 10.1007/s00360-017-1129-y

[ref75] Urbina MA , Schulte PM, Bystriansky JS, Glover CN (2013) Differential expression of Na^+^, K^+^-ATPase α-1 isoforms during seawater acclimation in the amphidromous galaxiid fish *Galaxias maculatus*. J Comp Physiol B 183: 345–357. 10.1007/s00360-012-0719-y. 23142926

[ref76] Vaz PG , Kebreab E, Hung SSO, Fadel JG, Lee S, Fangue NA (2015) Impact of nutrition and salinity changes on biological performances of green and white sturgeon. PloS One 10: e0122029. 10.1371/journal.pone.0122029. 25830227 PMC4382339

[ref77] Wood CM (1991) Acid-base and ion balance, metabolism, and their interactions, after exhaustive exercise in fish. J Exp Biol 160: 285–308. 10.1242/jeb.160.1.285.

[ref78] Wood CM , Turner JD, Graham MS (1983) Why do fish die after severe exercise? J Fish Biol 22: 189–201. 10.1111/j.1095-8649.1983.tb04739.x.

[ref79] Yang W-K , Chung C-H, Cheng HC, Tang C-H, Lee T-H (2016) Different expression patterns of renal Na*+*/K*+*-ATPase α-isoform-like proteins between tilapia and milkfish following salinity challenges. Comp Biochem Physiol B 202: 23–30. 10.1016/j.cbpb.2016.07.008. 27497666

[ref80] Zeidler R , Kim HD (1977) Preferential hemolysis of postnatal calf red cells induced by internal alkalinization. J Gen Physiol 70: 385–401. 10.1085/jgp.70.3.385. 19557 PMC2228469

[ref81] Zydlewski J , Wilkie MP (2013) Freshwater to Seawater Transitions in Migratory Fishes. In SD McCormick, AP Farrell, CJ Brauner, eds, Fish Physiology - Euryhaline Fishes. Cambridge, CA: Academic Press, pp. 253–326

